# Molecular processes involved in B cell acute lymphoblastic leukaemia

**DOI:** 10.1007/s00018-017-2620-z

**Published:** 2017-08-17

**Authors:** Camille Malouf, Katrin Ottersbach

**Affiliations:** MRC Centre for Regenerative Medicine, The University of Edinburgh, 5 Little France Drive, Edinburgh, EH16 4UU UK

**Keywords:** B cell acute lymphoblastic leukaemia, BCR-ABL1, MLL-AF4, ETV6-RUNX1, E2A-PBX1, Paediatric leukaemia

## Abstract

B cell leukaemia is one of the most frequent malignancies in the paediatric population, but also affects a significant proportion of adults in developed countries. The majority of infant and paediatric cases initiate the process of leukaemogenesis during foetal development (in utero) through the formation of a chromosomal translocation or the acquisition/deletion of genetic material (hyperdiploidy or hypodiploidy, respectively). This first genetic insult is the major determinant for the prognosis and therapeutic outcome of patients. B cell leukaemia in adults displays similar molecular features as its paediatric counterpart. However, since this disease is highly represented in the infant and paediatric population, this review will focus on this demographic group and summarise the biological, clinical and epidemiological knowledge on B cell acute lymphoblastic leukaemia of four well characterised subtypes: t(4;11) MLL-AF4, t(12;21) ETV6-RUNX1, t(1;19) E2A-PBX1 and t(9;22) BCR-ABL1.

## Introduction

Cancer is the top fatal disease in the paediatric population and now shares the first place in adults with heart disease [1]. It is a multifactorial disease that is influenced by genetics (e.g. chromosomal translocations, point mutations and single nucleotide polymorphisms), lifestyle (e.g. tobacco consumption and obesity), exposure to environmental factors (e.g. sunlight, radiation and infectious agents) and aging [2–4]. Over a lifetime, humans acquire a combination of genetic insults. Depending on the genetic insults and the cell-of-origin, this translates into the development of a sporadic cancer (~90% of cancers).

The scientific community has noted a continued increase of sporadic cancers in the paediatric population since the 1970s. This increase is strikingly restricted to developed countries, suggesting that our lifestyle plays a significant role in cancer development. Epidemiological factors that have been linked to an increased risk of developing paediatric B cell acute lymphoblastic leukaemia (B-ALL) include pesticides [5], magnetic exposure [6] and an overstimulation of the immune response during the first years of life (population-mixing and delayed-infection hypothesis) [7–9]. B-ALL accounts for 26% of cancers diagnosed in children aged 0–14 years old [10], making it one of the most prevalent cancers in the paediatric population. This disease leads to the differentiation arrest of B lymphoid cells at an immature stage (progenitor or precursor), followed by an uncontrolled proliferation and accumulation of leukaemia blasts in the bone marrow. These pro/pre-B leukaemia blasts will migrate from the bone marrow to the peripheral circulation to infiltrate the spleen, liver, thymus, lymph nodes and central nervous system. This hijack of the haematopoietic system by leukaemia blasts also compromises the production of other essential blood cell types for oxygen transport (erythrocytes) or coagulation (platelets). This leads to major insufficiencies in the patient’s immunological and physiological system, which ultimately results in death. Early signs of the disease include fatigue, loss of appetite, bone pain, swelling in the abdomen (due to splenomegaly and hepatomegaly) and swelling of the lymph nodes. Acute leukaemia has a rapid progression (weeks) compared to chronic leukaemia (months).

Therapies for B-ALL have greatly improved since the 1970s when the survival rate was only 10% [11]. Today, at least 90% of paediatric patients and 40% of adult patients will recover from this disease [12]. This treatment success is explained by breakthroughs in understanding the molecular mechanisms of leukaemogenesis, leading to the development of efficient and specific therapies and the use of stem-cell transplantation. The biology of B-ALL is determined mainly by the first genetic hit, which is usually a change in chromosome numbers (hyperdiploidy or hypodiploidy) or a chromosomal translocation (e.g. t(4;11) MLL-AF4 or t(12;21) ETV6-RUNX1) (Table 1). In paediatric patients, this genetic insult can have an in utero origin that can be traced back to birth by analysing the Guthrie cards which contain newborn blood spots [13]. Increasing evidence suggests that additional genetic events are required for leukaemia development, which will be described in each section. Furthermore, patients at relapse show deletions at specific loci (e.g*. CDKN2A/B*, *ETV6*, *IKZF1*, *NR3C1*) [14]. Some patients also acquire mutations in *NT5C2*, leading to drug-resistance [15], and in *CREBBP* which impairs the acetylation and transcriptional regulation of CREBBP-target genes [16].

**Table 1:** Subtypes of B cell acute lymphoblastic leukaemia and their frequencies within specified age groups

Cytogenetic abnormality	Frequency (%)			Prognosis
	Infant (<1 year old)	Children (2–18 years old)	Adult (>18 years old)	
t(4;11)(q21;q23) *MLL*-*AF4*	50–85	2–20	10	Poor
t(12;21)(p13;q22) *ETV6*-*RUNX1*	Rare	15–12	2	Good
*ETV6*-*RUNX1*-like	Rare	6	Rare	Good
t(1;19)(q23;p13) *E2A*-*PBX1*	Rare	2–6	3	Good
t(9;22)(q34;q11.2) *BCR*-*ABL1*	Rare	1–3	25	Poor
*BCR*-*ABL1*-like	Rare	15–20	30–40	Poor
*DUX4*-rearranged	Rare	8	Rare	Good
Hyperdiploidy	Rare	20–30	7	Good
Hypodiploidy	Rare	1–2	2	Poor
Trisomy 4 and 10	Rare	20–25	Rare	Very good
Intrachromosomal amplification of chromosome 21 (iAMP21)	Rare	2–3	11	Poor

This review will explore the biological mechanisms that are affected by four chromosomal translocations: t(4;11) MLL-AF4, t(12;21) ETV6-RUNX1, t(1;19) E2A-PBX1 and t(9;22) BCR-ABL1. The first three are associated with perturbations in the gene expression profile, whereas t(9;22) BCR-ABL1 hijacks signalling pathways. Since the members of each translocation are involved in normal haematopoiesis, we will also describe their role in a physiological situation. We have chosen to focus on these four subclasses as they cover around 40% of B-ALL patients in children and adults (and 80% in infants) and they have been well studied in patients and animal models. However, for completeness’ sake, we will also briefly mention other subtypes: including hyperdiploidy, hypodiploidy, trisomy 4 and 10 and intraamplification of chromosome 21. In addition, the therapeutic avenues currently available in the clinic will be discussed and potential new agents highlighted that are in clinical trials (Table 2).

**Table 2:** Current clinical trials in B cell acute lymphoblastic leukaemia

Cytogenetic abnormality	Risk group	Therapeutic target (drug)	Clinical trial (as of July 2017)
t(4;11)(q21;q23) *MLL*-*AF4*	High	I-BET inhibitor (I-BET151 and I-BET762)	NCT01943851 (phase I, recruiting)
		DOT1L inhibitor (EPZ5676)	NCT01684150 (phase I, complete) NCT02141828 (phase I, complete)
		Flavopiridol	NCT00278330 (phase I, complete)
		Menin–MLL interaction (MI-2, MI-2-2)	Preclinical
		LEDGF–MLL interaction (CP65 small peptide)	Preclinical
		WDR5–MLL interaction (MM-401)	Preclinical
t(1;19)(q23;p13) *E2A*-*PBX1*	Standard	mTOR inhibitor (rapamycin or analog)	NCT00874562 (phase I, complete) NCT00081874 (phase I/II, complete)
		p110δ inhibitor (idelalisib)	NCT01539512 (phase III, complete)
		SRC inhibitor (dasatinib)	NCT00438854 (phase I, complete)
		LCK inhibitor (A-770041)	Preclinical
		SYK inhibitor (P505-15)	Preclinical
		AuroraB inhibitor (barasertib-HQPA)	NCT00926731 (phase I, complete) NCT01019161 (phase I, complete)
t(9;22)(q34;q11.2) *BCR*-*ABL1*	High	PI3K/mTOR inhibitor (PI-103)	None (fast in vivo metabolism)
		PI3K/mTOR inhibitor (NVP-BEZ235)	NCT01756118 (phase I, active)
		Farnesyl transferase inhibitor (lonafarnib)	NCT00034684 (phase II, complete)
		Mutant forms of BCR-ABL (ABL001/asciminib, nilotinib)	NCT01528085 (phase II, active) NCT03106779 (phase III, not yet recruiting)
		Native form of BCR-ABL (imatinib)	NCT00025415 (phase I, complete)
		JAK2 inhibitors (ruxolitinib)	NCT01251965 (phase I-II, complete) NCT02723994 (phase II, recruiting)
*BCR*-*ABL1*-like	High	Dasatinib (*ABL1*, *ABL2*, *CSF1R*, *PDGFRB* fusion genes)	NCT00438854 (phase II, complete)
		Ruxolitinib (*EPOR* and *JAK2* rearrangements)	NCT01251965 (phase I/II, complete) NCT02723994 (phase II, recruiting)
		Crizotinib (*ETV6*-*NTRK3*)	NCT02551718 (ex vivo, recruiting)
Hypodiploidy	High	PI3K inhibitor (GDC-0941)	NCT00876122 (phase I, complete)
		PI3K+ mTOR inhibitor (NVP-BEZ235)	NCT01756118 (phase I, active)

## The fast and the furious: t(4;11)(q21;q23) MLL-AF4

### Clinical features of MLL-AF4+ patients

The rearrangement of the *MLL* gene (11q23) is a common genetic event in haematological malignancies [17]. It is present in around 10% of ALL and 5% of acute myeloid leukaemia (AML). There are more than 80 genes that can form chromosomal translocations with the *MLL* gene in leukaemia, with *AF4*, *AF9* and *ENL* amongst the most common. *MLL*-rearrangements are found in both paediatric and adult ALL, but the prognosis is poorer in childhood ALL regardless of the age at diagnosis [18]. Around 50% of patients diagnosed with pro-B ALL aged 6 months or less harbour the t(4;11) MLL-AF4 chromosomal rearrangement [19]. The frequency of t(4;11) MLL-AF4 ALL decreases in older infants (10–20%) and drops to just 2% in children. In adult B-ALL, it accounts for around 10% of cases.

t(4;11)+ leukaemia is associated with an increased leukocyte count due to an accumulation of leukaemia blasts with a phenotype similar to progenitor B cells (negative for CD10) [18]. The leukaemia blasts often co-express haematopoietic progenitor markers (CD34), B lymphoid markers (CD19, CD22, CD79a, Tdt), myeloid markers (CD15, CD65) and components of the leukocyte antigen complex (HLA-DR) [20]. They are negative for CD24, IgM, CD13 and CD33. A significant proportion of adult and paediatric patients express the neural glial marker NG2 on blast cells [21, 22], which is also detected in t(12;21) ETV6-RUNX1 B-ALL and t(9;11) MLL-AF9 AML [23]. Interestingly, the NG2 antigen is not expressed in haematopoietic stem cells derived from adult bone marrow or neonatal cord blood, but is expressed in more mature haematopoietic progenitor cells [23, 24].

An interesting feature of t(4;11)+ infant patients is the expression of MLL-AF4 in a subset of bone marrow mesenchymal stem cells (MSC) [25]. This was not observed in other types of B-ALL and seems to be unique to this group of patients. It suggests that the initial translocation occurred in a precursor for both the haematopoietic and the stromal compartment. To investigate the impact of MLL-AF4 expression in early development, Bueno et al. induced the expression of this fusion gene through a lentiviral vector in human embryonic stem cells (hESC) with expression levels similar to those found in patient-derived cell lines [26]. MLL-AF4 induced an upregulation of HOX gene expression and enhanced the formation of haemogenic precursor cells (CD45^−^CD31^+^CD34^+^). This was accompanied by a diminished haematopoietic differentiation and an increased endothelial cell fate. Therefore, MLL-AF4 does not seem to favour haematopoietic commitment during the mesodermal to haematopoietic transition; however, MLL-AF4 expression in the stromal compartment may increase niche support for the cell-of-origin and the leukaemia blasts.

### MLL, a regulator of transcription essential for embryonic and adult haematopoiesis

MLL, also known as KMT2A, is an H3K4 methyltransferase that catalyses histone H3 Lys4 methylation through its SET domain. This epigenetic mark is associated with actively transcribed promoters and open chromatin. MLL directly interacts with the nuclear protein Menin (MEN1), which is essential for MLL-mediated transcription and for MLL-AF4-mediated transformation [27, 28]. Accordingly, MEN1 inhibitors were shown to be detrimental to oncogenic transformation by MLL-fusion genes, independent of the MLL partner gene [29, 30]. Inhibitors of WDR5 and LEDGF, two partners of MLL, can also disrupt MLL activity and the oncogenic potential of MLL-AF4 [31–33].

MLL plays a vital role during embryonic development by regulating HOX gene expression, which is crucial for the specification of segment identity [34]. *Mll* haploinsufficiency in mice leads to major disorders in the cervical, lumbar and thoracic regions. Hence, Mll is critical for pattern formation and proper development of the embryo. A complete knock-out of *Mll* in mice leads to death at embryonic day (E)10.5 because of dysplasia in the branchial arch and aberrant segment boundaries of spinal ganglia and somites [35]. E10.5 is also the developmental time-point when the first definitive haematopoietic stem cells (HSCs) emerge in the aorta-gonads-mesonephros (AGM) region in a process that depends on Runx1, a transcription factor linked to pre-B ALL ([36, 37] and see below). Subsequent work from the Korsmeyer group has shown that Mll is important for maintaining haematopoietic potential throughout embryonic development. Mll is essential for the haematopoietic colony-forming potential and proliferation of haematopoietic progenitors in the E10.5 yolk sac [38], the tissue in which haematopoietic cells are first detected [39]. Mll continues to have a role in maintaining the haematopoietic potential at later stages in the E12.5 foetal liver and yolk sac [40]. Furthermore, *Mll*-deficient embryonic stem (ES) cells cannot reconstitute haematopoietic organs in chimeric animals, leading to a lack of haematopoietic potential in the foetal liver [41]. Importantly, *Mll*-null AGMs are devoid of HSCs [41]. Mll also has a cell-intrinsic role in the self-renewal of HSCs from the foetal liver and adult bone marrow since its deletion leads to an impaired engraftment in competitive transplants [42]. Hence, Mll is a gene that is essential to maintain the haematopoietic potential during both embryonic and adult development.

### AF4, a mediator of RNA elongation essential to lymphopoiesis

AF4 is a member of the AF4/FMR2 family that contains four members: AFF1/AF4, AFF2/FMR2, AFF3/LAF4 and AFF4/AF5q31. Most members, with the exception of AFF2/FMR2, can translocate with the *MLL* gene and participate in the development of ALL or AML. AF4 is part of the AEP complex, which includes other members of the AF4/FMR2 family (AF5Q31), the ENL family (ENL and AF9) and the p-TEFB elongation factor. The AEP complex is important for releasing the paused RNA polymerase II, which initiates RNA elongation. As mentioned previously, *MLL* can fuse to more than 80 different partner genes in haematological malignancies, most of which are members of the AEP complex. Some members of this family (AFF2/FMR2, AFF3/LAF4 and AFF4/AF5q31) also localise to nuclear speckles which are structures containing pre-mRNA splicing factors [43]. Those structures contain the regulatory subunit cyclin T1 and the catalytic domain CDK9, which together form the p-TEFB elongation factor. P-TEFB can be inactivated by flavopiridol [44], which has completed its phase I clinical trial for recurrent B-ALL in adults (NCT00278330). Hence, some members of the AF4/FMR2 family can also participate in the splicing of messenger RNA, and this process could be tightly associated with RNA elongation. However, AF4 does not localise to nuclear speckles, so it is unlikely that the MLL-AF4 fusion gene can deregulate this pathway.

Af4 is ubiquitously expressed, but its level of expression is higher in the lymphoid compartment and placenta [45, 46]. *Af4* ^−/−^ knockout mice show a severe defect in lymphopoiesis compared with *Af4* ^*+/−*^ mice, as evidenced by reduced numbers of B and T cells in the main adult haematopoietic sites such as the bone marrow, spleen and thymus [47]. AF4 can also promote the expression of CD133, a cell surface marker of hematopoietic and cancer stem cells [48]. The immortalisation of myeloid progenitors by the MLL-AF4 fusion gene requires the AF4-binding platform (pSER domain) as shown in colony replating assays [49]. AF4 is also important for recruiting selectivity factor 1 (SL1), which is a specific pSER domain binder, and this ensures the loading of TBP to the TATA box [50]. This study provides new evidence for a transactivation role of AF4 in the leukaemogenesis process. The N-terminal part of AF4 can bind the pTEFb complex, but also recruit TFIIH and MEN1 [51]. This is interesting since the AF4-MLL reciprocal fusion gene has also been implicated in B-ALL development. This will be discussed later in this section.

### The biology of t(4;11) MLL-AF4 infant leukaemia

Cancer development is a disease that is normally associated with the acquisition of an array of mutations throughout a lifetime. Paediatric ALL, however, has one of the lowest mutation rates, which is estimated at approximately 0.2–0.4 mutations per MegaBase [52]. Since this disease is usually initiated in utero at a developmental stage where the chromatin is more open and accessible than in adults [53], it is possible that all the factors required by MLL-AF4 to initiate disease are already active. Whole-genome, exome and targeted DNA sequencing studies in t(4;11) infant patients have confirmed the absence of cooperating mutations in this sub-type of leukaemia [54]. A recent study using ultra deep sequencing of 36 paediatric t(4;11) MLL-AF4 ALL found that 63.9% of them had a mutation in *NRAS* or *KRAS* that could lead to its hyperactivity [55]; however, experimental evidence suggests that these may influence the timing of disease onset and malignant cell migration, but are not required for disease initiation [56, 57], as further discussed below. It is thus clear that t(4;11)+ pro-B ALL has a lower mutation rate than other subtypes of B cell leukaemia, suggesting that MLL-AF4 alone is sufficient to initiate disease.

Several attempts have been made to generate a mouse model for MLL-AF4+ leukaemia. First, in 2006, Chen et al. developed a constitutive knock-in mouse, in which human *AF4* was inserted into the mouse *Mll* locus [58]. They obtained some heterozygous *Mll*-*AF4* knock-in animals, despite noticeable embryonic lethality. These were smaller at birth compared to their wild-type littermates, presented with a higher white blood cell count in the peripheral blood, increased levels of neutrophils and a bias toward the B lymphoid lineage [58]. Cells from *Mll*-*AF4* knock-in mice produced more pro-B cell colonies in methylcellulose assays compared with their wild-type littermates, and could serially replate for three rounds. This suggested an increased self-renewal when Mll-AF4 is expressed and an immortalisation of the pro-B cells. However, when those cells were placed in liquid culture, they could not survive for more than two months, implying that the transformation process was not completed. The *Mll*-*AF4* knock-in mice did develop haematological malignancies (lymphoid and myeloid hyperplasia, B cell lymphoma and myeloid malignancy) with a median time of 570 days. Therefore, MLL-AF4 on its own can lead to a haematological malignancy, but this model did not recapitulate the human disease.

Because of the high embryonic lethality rate observed in constitutive *Mll*-*AF4* knock-in mice, Metzler et al. developed a conditional knock-in model based on the invertor technology and the use of specific Cre recombinases [59]. Using Cre recombinase expressed under the control of specific lymphocyte promoters, they successfully generated mice that expressed Mll-AF4 in B lymphocytes (*CD19*-Cre), T lymphocytes (*Lck*-Cre) or both (*Rag*-Cre). In all cases, the mice developed large diffuse B cell lymphomas with a latency of 317–475 days, which was shorter than the previous Mll-AF4 knock-in mice. Even though the phenotype of the disease was still dissimilar to the human counterpart, it revealed a bias of MLL-AF4-driven disease towards the B lineage even when targeted to T cells.

In 2008, Krivtsov et al. published their work with a new conditional knock-in mouse model of *Mll*-*AF4*, which used the *Mx1*-Cre system to activate the expression of Mll-AF4 postnatally after an induced interferon response [60]. Following multiple intraperitoneal injections of polyinosinic/polycytidylic acid (polyI:C) to initiate the expression of Mll-AF4, 36% of the mice developed a haematological malignancy with a latency of 152 days and a disease phenotype that was similar to pre-B ALL, while 27% developed AML. It thus appeared that all of these mouse models were missing a crucial element required for the development of a disease more representative of the human leukaemia. One of the hypotheses was that a human physiological context was required. With the emergence of humanized mice that have a compromised immune system and can, therefore, tolerate human haematopoietic cell transplants (as reviewed in Ref. [61]), it is possible to use human haematopoietic cells to enhance our understanding of pro-B ALL.

Thus, Montes et al. isolated human cord blood CD34^+^ haematopoietic stem and progenitor cells and transduced them with a lentiviral vector containing the human *MLL*-*AF4* cDNA [62]. The authors found an increased engraftment of human cord blood cells in NSG mice when MLL-AF4 was expressed. This was accompanied by an increase in HOXA9 expression. The clonogenic potential of the cells was slightly enhanced in myeloid-forming conditions, but the mice did not develop any haematological malignancies. This study suggested that human cord blood cells might represent a stage of development that is too late to recapitulate the leukaemogenesis happening in patients. In 2016, Lin et al. used an alternative approach [63] and created a retroviral construct that contains the human *MLL* and the mouse *Af4* gene parts. Compared to the human MLL-AF4 construct, their hybrid produced a higher retroviral titer. They transduced the lineage negative fraction (Lin-) of mouse adult bone marrow, which contains all of the haematopoietic stem and progenitor cells, and cultured these in myeloid or lymphoid conditions. Following transplantation of the cultured cells, the recipients developed AML, irrespective of the culture conditions. This may at least partly be due to the Lin− fraction being mostly enriched in mature myeloid progenitors such as GMP and CMP, which would be preferentially targeted. Interestingly, when they transduced human CD34^+^ cells derived from cord blood and transplanted these into NSG recipients, the mice developed pro-B ALL. The authors propose that the development of pro-B ALL requires a human haematopoietic context. It does not, however, seem to require a prehaematopoietic or hemangioblast precursor, or additional events like the reciprocal fusion gene AF4-MLL.

Infant t(4;11) MLL-AF4 pro-B ALL has an in utero origin [13]. Therefore, to understand the first stages of the disease, it is important to initiate the expression of MLL-AF4 in embryonic haematopoietic cells which have unique and different properties than adult haematopoietic cells (as reviewed in Ref. [64]). Barrett and Malouf et al., therefore, used the conditional Mll-AF4 invertor mice developed by Terry Rabbitts and two different Cre lines to target the expression of Mll-AF4 to foetal haematopoietic cells: *Vav*-Cre (definitive haematopoietic cells) and *Vec*-Cre (haemogenic endothelium) [65]. The experimental set-up allowed targeting of all the haematopoietic cells formed during the definitive wave of haematopoiesis, but also the haemogenic endothelium that gives rise to HSCs in the AGM (E10.5 of development), lymphoid progenitors (E9.5) and erythro-myeloid progenitors (E8.5) in the yolk sac [66, 67]. The effect of Mll-AF4 on emerging blood cells was then analysed throughout haematopoietic development in the embryo. Blood cells formed in the AGM and placenta (E11-E12) showed a slight lymphoid bias in colony assays, but their engraftment was similar to control cells, suggesting that the transformation process is not initiated at those early stages of definitive haematopoiesis. At later stages in the foetal liver (E12–E14), when haematopoiesis has established itself, the engraftment potential of foetal liver cells was significantly enhanced upon Mll-AF4 expression and the B lymphoid bias became stronger in colony-forming assays. This was also accompanied by a higher production of myeloid colonies. Most mice developed B cell lymphomas with a latency of 437 days (*Vec*-Cre) or 556 days (*Vav*-Cre). This study also shed light on the cell-of-origin of infant pro-B ALL, which will be discussed in the next section.

### The lymphoid-primed multipotent progenitor (LMPP) as a potential cell-of-origin

Previous studies have suggested that leukaemia originates in a haematopoietic progenitor that has a lineage-restricted differentiation potential, but has retained stem cell-like features. Those stem-cell properties confer higher self-renewal and quiescence, which makes them resistant to chemotherapeutic agents that can target proliferative leukaemia blasts [68, 69]. Over the past few years, studies have shed light on the cell-of-origin, but the debate is still open, especially for B-ALL.

In one study, Cox et al. [70] found that the human CD34^+^CD19^−^ and CD34^+^CD10^−^ fractions derived from the bone marrow of B cell leukaemia patients aged 1–16 years old could engraft immunocompromised NOD/SCID mice and lead to the development of a leukaemia with a phenotype similar to patients. Long-term cultures of unsorted bone marrow became strongly enriched for CD34^+^CD19^−^ and CD34^+^CD10^−^ fractions with time, with a concomitant loss of the CD19^+^ and CD10^+^ fraction, which also correlated with their lack of engraftment in xenotransplantation. Therefore, they concluded that the target cells for leukaemia initiation in B-ALL are immature haematopoietic progenitors that have not committed to the B cell lineage.

Le Viseur et al. sorted leukaemia cells from B-ALL patients according to the expression of CD34 and CD19, followed by xenotransplantation [71]. CD34^+^CD19^−^, CD34^+^CD19^+^ and CD34^−^CD19^+^ fractions all engrafted robustly and could initiate leukaemia development with a phenotype similar to what was observed in patients and a mean latency of 11.7 weeks.

Aoki et al. reported that the CD34^+^CD38^−^CD19^−^CD33^−^ bone marrow fraction of infant t(4;11) MLL-AF4 pro-B ALL patients contained normal haematopoietic stem and progenitor cells since they gave rise to normal myelopoiesis and lymphopoiesis in xenotransplantation [72]. The injected cells did not initiate leukaemia or expressed MLL-AF4. However, xenotransplantation of CD34^+^CD38^+^CD19^+^ or CD34^−^CD19^+^ cells did lead to leukaemia development, and those cells expressed MLL-AF4. The expression of CD19 seems important for the leukaemia potential in xenotransplantation, but as mentioned previously, CD19^−^ cells derived from patients can engraft and initiate leukaemia development in other studies [70, 71]. At a similar time, Bardini et al. found that the expression of CD34 and NG2 cannot differentiate leukaemia-initiating cells from non leukaemia-initiating cells [73]. They also found some Ig/TCR rearrangement in the xenografted cells, suggesting that the founder clone could be a haematopoietic progenitor that has already undergone some rearrangement (CD19^+^). There is also a possibility that the founder clone could be a cell that has no mature antigen receptor rearrangement (e.g. IGH, IGK-Kde, TCRD, TCRG) rearrangement (CD19^−^), which was observed in leukaemia cells from t(4;11) pro-B ALL patients [74]. The question of whether transformation begins before the commitment to the lymphoid lineage in patients, therefore, requires further investigation.

Using an inducible mouse model in which Mll-AF4 was targeted to the first definitive haematopoietic cells during development, Barrett and Malouf et al. found an increased B lymphoid output in the E12–E14 foetal liver during embryonic development compared to the control littermates [65]. They isolated two fractions: HSC/MPP (Lineage^−^ Sca1^+^ ckit^+^ Flt3^−^ IL7R^−^; stem cell and immature progenitors) and LMPP (Lineage^−^ Sca1^+^ ckit^+^ Flt3^+^) and observed a notable increase in the formation of B cell colonies from both populations upon expression of Mll-AF4. These colonies were bigger in size compared to colonies formed by control cells and contained mostly pro-B cells (AA4.1^−^ckit^+^B220^+^CD19^+^CD43^+^CD24^+^IgM^−^) that could form colonies in replating assays. The clonogenic potential of the LMPP was around fourfold higher compared to the HSC/MPP fraction. All together, these results confirm that leukaemogenesis is initiated in utero and that Mll-AF4 confers stem-cell like properties to embryonic haematopoietic progenitors. This study also suggests that the foetal liver LMPP could be the cell-of-origin of t(4;11) MLL-AF4 leukaemia [65].

### MLL-AF4 is a positive regulator of clonogenicity and survival

To gain further insights into the role of MLL-AF4 in leukaemia, scientists have used various human and mouse cell lines to assess its effect on survival, proliferation and clonogenic potential. Caslini et al. developed a tetracycline-inducible system of MLL-AF4 expression in U937 human monocyte cells and observed that an increased expression of MLL-AF4 leads to an inhibition of proliferation [75]. This phenotype was similar to the overexpression of MLL-AF9 [75]. By contrast, Thomas et al., using siRNA technology to inhibit the expression of MLL-AF4 in SEM and RS4;11 cells, observed that the inhibition of MLL-AF4 had a negative effect on the clonogenic potential and proliferation of leukaemia cells [76]. This was accompanied by a decreased engraftment in SCID mice and an upregulation of CD133, suggesting that the cells had started to differentiate and were losing their self-renewal potential. These seemingly contrasting results suggest that either the cell context can impact on the transformation process mediated by MLL-AF4 or maintaining the right level of MLL-AF4 expression is crucial, with knockdown and overexpression both having a negative impact on cell proliferation. The former interpretation is supported by recent work from the Mulloy group who revealed an impaired ability of MLL-Af4 to transform myeloid cells [77]. This lymphoid preference is driven by the Af4 partner and requires a lymphoid cellular context. The expression of MLL-AF4 can also be modulated by specific microRNAs that can recognize the 3′UTR of AF4, notably miR-143 [78]. The miR-143 locus is highly methylated in MLL-AF4+ leukaemia cell lines, leading to its downregulation and MLL-AF4 escaping microRNA-mediated regulation. Restoring the expression of miR-143 in MLL-AF4+ leukaemia cells leads to an increase in apoptosis and a decrease in cell proliferation. Hence, microRNAs can influence leukaemogenesis and bring an additional layer of complexity to our understanding of pro-B ALL.

### Contributors to t(4;11) MLL-AF4 leukaemia

All the mice that express MLL-AF4 do develop a haematological malignancy; however, they do not recapitulate the human disease that has a fast progression and produces leukaemia blasts with a pro-B cell phenotype. These models suggest that additional events or factors are necessary, including changes in gene expression which may not be present in the murine context.

### FLT3 activity in infant leukaemia

The activity of FLT3 is increased in t(4;11) MLL-AF4 pro-B ALL and other cytogenetic groups including t(12;21) ETV6-RUNX1 and t(9;22) BCR-ABL1 [79]. This can result from mutations in the tyrosine kinase domain (TKD) or a tandem duplication of the gene, but is usually associated with an increased expression of FLT3, commonly as a result of an upregulated HOXA9/MEIS1 program [80]. The prognosis of t(4;11) MLL-AF4 leukaemia can be predicted with FLT3 expression. High expression of FLT3 is associated with a bad prognosis, a shorter overall survival and a shorter disease-free survival, while low expression of FLT3 is associated with a better prognosis. To model the effect of FLT3, Montes et al. transduced human CD34^+^ cord blood cells with MLL-AF4 and either an overactive FLT3 (FLT3-TKD) or a wild-type FLT3 [81]. Using xenotransplantation, they assessed the development of leukaemia upon activation of FLT3. Even though the mice did not develop leukaemia, they observed a transient expansion of CD34^+^ cord blood cells expressing MLL-AF4 upon FLT3 activation.

### AF4-MLL: the reciprocal fusion gene

The reciprocal fusion gene AF4-MLL is present in approximately 80% of patients diagnosed with t(4;11) MLL-AF4 leukaemia [82]. Similar to MLL-AF4, it contains cleavage sites for Threonine-Aspartase 1 (Taspase 1) which are important for its activation and its resistance to proteosomal degradation. AF4-MLL can associate with proteins that modulate the activity of RNA polymerase II promoters (e.g. P-TEFB kinase) and alter the histone methylation signature by recruiting H3 arginine and lysine methyltransferases, such as CARM1 and DOT1L, respectively, leading to an increased H3 methylation signature [83]. Therefore, both MLL-AF4 and AF4-MLL can increase H3K79, H3R2, H3R17 and H3R26 methylation marks. Additionally, AF4-MLL can increase the H3K4 methylation mark and extend the activation of gene expression. AF4-MLL can also interact with the C-terminal portion of AF4, suggesting that MLL-AF4 can be recruited to the same transcriptional complex. Neither MLL-AF4 nor AF4-MLL seem to affect the efficiency of DNA damage repair [84]. MLL-AF4 and AF4-MLL can modulate the transcription of the *ALOX5* gene through the activation of transcription and elongation, respectively [85]. The *ALOX5* gene encodes the 5-lipoxygenase enzyme, which is essential for the formation of pro-inflammatory leukotrienes. These metabolites of arachidonic acid can activate the immune response and help to establish a tumour microenvironment by acting both on cancer cells and stromal cells (as reviewed in Ref. [86]). They have also been linked to proliferation, as an increased production of leukotriene B_4_ in B cell chronic lymphocytic leukaemia was shown to enhance DNA synthesis [87]. Interestingly, it was shown that AF4-MLL can lead to leukaemia, even in the absence of MLL-AF4 [88]. Therefore, AF4-MLL appears to be important for leukaemia development, but its absence in 20% of patients implies it is not always essential.

### NRAS and KRAS

The mutation of *NRAS* and *KRAS* in t(4;11) MLL-AF4 pro-B ALL has been reported in the literature despite the low mutation burden in this subtype of leukaemia [89]. MLL-AF4 can also activate the expression of the ELK-1 transcription factor, which is a downstream effector of KRAS activity [90]. This prompted scientists to study its role in leukaemogenesis. Crossing a transgenic MLL-AF4 mouse line with a transgenic *Kras* mouse line [57] resulted in the mice developing B cell lymphoma or leukaemia characterised by the accumulation of B220^+^CD43^+^CD19^+^ blasts, splenomegaly and infiltration of malignant cells in the lungs. The latency was significantly reduced by the presence of *Kras* mutations. Prieto et al. used xenotransplantation to assess the contribution of KRAS activation to the transformation of CD34^+^ cord blood cells expressing MLL-AF4 [56]. They found that the expression of KRAS favoured the migration of cells to extramedullary organs, but the mice did not develop leukaemia. A non-essential role for RAS signalling is also supported by the observation made in a recent large-scale sequencing study in which activating mutations in the RAS pathway were found to be mostly sub-clonal and often lost at relapse [54].

### Transcriptional deregulation as a hallmark of MLL-rearranged leukaemia

The expression of MLL-AF4 is associated with an ectopic H3K79 methylation profile that is crucial for the transformation process in infant pro-B leukaemia and requires the aberrant recruitment of the DOT1L enzyme [60, 91]. Importantly, this is now explored as a potential therapeutic target [91, 92] with DOT1L inhibitors being currently tested in clinical trials. In addition, MLL-rearranged leukaemias have the highest DNA methylation index among all types of leukaemias [93].

The epigenetic abnormalities result in the upregulation in leukaemia blasts of an array of genes which are normally expressed in haematopoietic stem cells, such as HOXA9, MEIS1, HMGA2 and RUNX1 [37, 94–96]. Interestingly, *RUNX1* was not only determined to be a direct target of MLL-AF4, but the RUNX1 protein was also found to form a complex with the reciprocal AF4-MLL fusion protein [97]. Around 50% of infant t(4;11) MLL-AF4 patients show an upregulation of HOX genes, which in combination with MEIS1, participate in the immortalisation process mediated by MLL-rearranged genes.

The HMG/high mobility group family of transcription factors (HMGA1 and HMGA2) are ubiquitously expressed during embryonic development, but almost undetectable in adult tissues. They are chromatin-remodelling factors that bind AT-rich regions of the minor groove of DNA. HMGA2 expression is upregulated in MLL-AF4 leukaemia cell lines which leads to an increased cell proliferation that is dependent on the hypermethylation of the miR-let-7b promoter [98]. This MLL-AF4/let-7b/HMGA2 axis is sensitive to the demethylating agent 5-azacytidine. Studies have suggested that MLL-AF4 can modulate the expression of BCL-2 target genes, which are members of the intrinsic mitochondrial apoptosis pathway [97, 99, 100]. In fact, MLL-AF4 can increase the expression of BCL-2 and MCL-1 and repress the transcription of BIM through binding of the promoters [28]. Accordingly, the inhibition of MLL-AF4 in the t(4;11)+ SEM cell line led to a decrease in H3K79me2/3 methylation at the *BCL*-*2* locus and a decrease in H3K27Ac acetylation mark at the 3′ end [28, 100]. The expression of BCL-2 resulted in resistance to apoptosis, which could be reverted using a specific BCL-2-selective inhibitor ABT-199. Inhibition of MLL-AF4 also led to reduced binding of the PRC1 protein CBX8 to the *BIM* locus. Similar to the effects of MLL-AF4 inhibition, treatment of SEM cells with the DOT1L inhibitor EPZ5676 caused a loss of the histone marks and a decreased expression of BCL-2 and MCL-1, but not BIM.

**Fig. 1:**
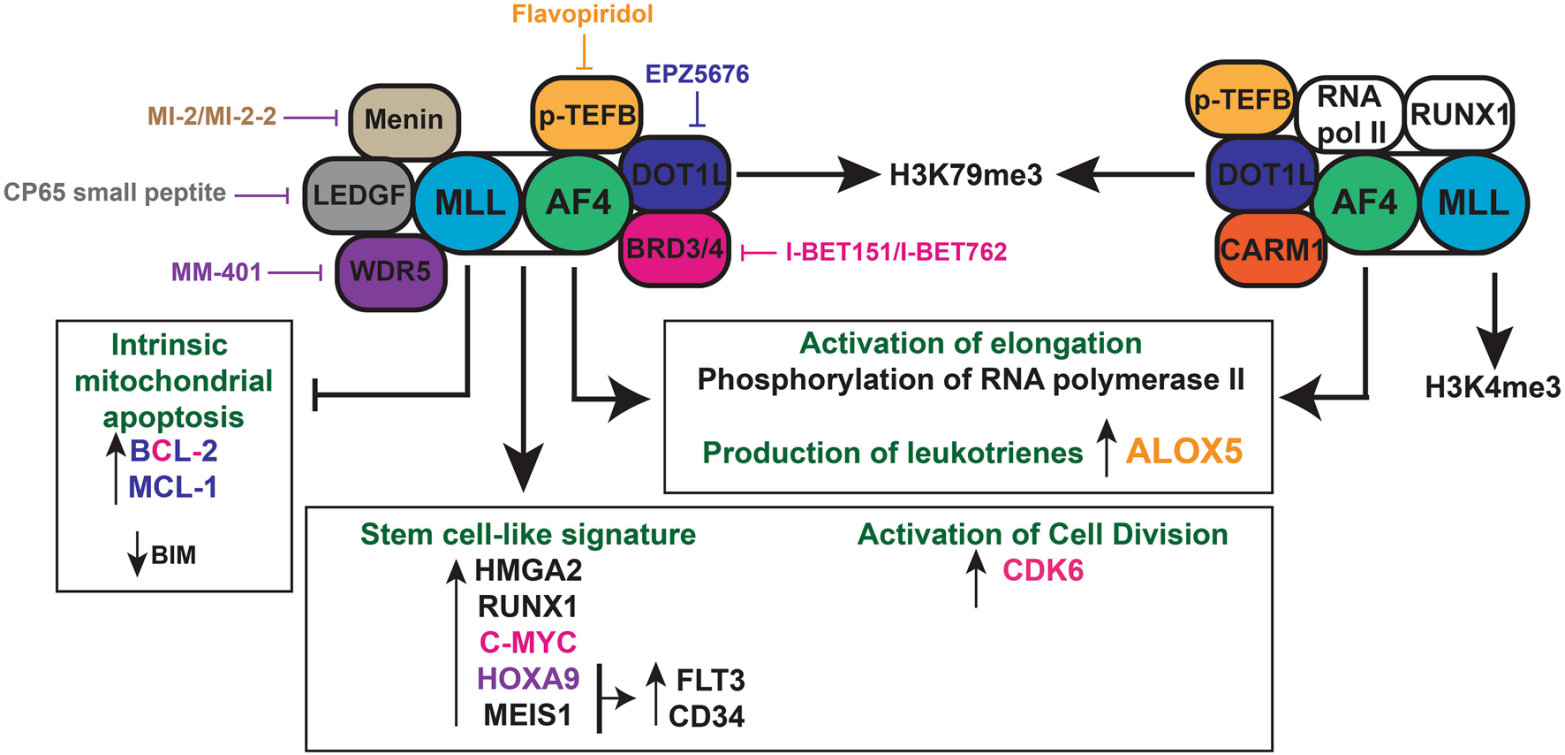
Biology of t(4;11) MLL-AF4 pro-B acute lymphoblastic leukaemia. The *MLL*-*AF4* fusion gene and *AF4*-*MLL* reciprocal fusion gene are shown with their main interaction partners. Both induce a deregulated epigenetic signature leading to an upregulation of stem-cell signature genes, positive regulators of cell division and inhibitors of apoptosis. The DOT1L inhibitor (EPZ5676), I-BET inhibitor (I-BET151), p-TEFB inhibitor (flavopiridol), WDR5 inhibitor (MM-401) and LEDGF inhibitor (CP65 small peptide) are potential therapeutic agents that target various members of the MLL-AF4 complex and its regulated genes

In 2011, Dawson et al. investigated the effect of an inhibitor of the BET family I-BET151 on MLL-rearranged leukaemia [101]. I-BET151 works by removing the BRD3/4, PAFc and SEC components from chromatin. They found that this inhibitor had a strong effect on the survival of MLL-rearranged leukaemia cells derived from patients, and this was partly due to the inhibition of transcription of BCL-2, the oncogene C-MYC and CDK6, the latter of which is required for the exit of haematopoietic stem cells from quiescence [102]. At the moment, phase I clinical trials are underway using I-BET inhibitors (NCT01943851) [103] and inhibitors of DOT1L (NCT01684150, NCT02141828) [104]. The inhibitors for menin, LEDGF and WDR5 are still in their preclinical phases [105]. The biological features and therapeutic avenues of t(4;11) MLL-AF4 pro-B ALL are highlighted in Fig. 1.

## The usual suspect: t(12;21) ETV6-RUNX1

### Clinical features of ETV6-RUNX1+ patients

Approximately 25% of patients diagnosed with paediatric pre-B ALL will present with the t(12;21) ETV6-RUNX1 translocation, which makes it the most frequent genetic insult [106]. The leukaemia blasts from pre-B ALL patients are CD10^+^, unlike t(4;11)+, and express CD19 and CD34 [107]. The t(12;21) translocation involves the fusion of two transcription factors essential for haematopoiesis: ETV6 and RUNX1. Both genes are expressed in HSCs and more mature haematopoietic progenitors such as LMPP, CLP, GMP and PreMegE [108]. This leads to the formation of a chimeric transcription factor that contains the protein–protein interaction domain of ETV6 (PNT), located on the N-terminal side, with almost the entire *RUNX1* gene. The ETV6-RUNX1 chimeric transcription factor does not contain the DNA-binding site of ETV6 (the ETS domain). t(12;21)+ patients have a good prognosis and the risk of relapse is low compared with t(4;11)+ patients. Nearly all ETV6-RUNX1+ pre-B ALL patients can be cured with the minimal residual disease/MRD-guided treatment regimen [109]. Similar to t(4;11) MLL-AF4, the t(12;21) ETV6-RUNX1 originates in utero. Studies in twins found that the genomic breakpoint of the *ETV6*-*RUNX1* chromosomal translocation was identical [110], suggesting that the pre-leukaemic clone reached the other twin via a shared placenta. However, only around 1% of newborns that harbour the t(12;21) will develop leukaemia [111], suggesting that the development of t(12;21) pre-B ALL requires additional mutations. It has been reported that RAG-mediated deletions is one of the main mutational processes [98]. This leads to chromosomal deletions in various genomic loci including *ETV6*, *PAX5*, *BTG1*, *LIMD1*, *NR3C2*, *ATM*, *AIM1* and *BLIMP1* [112, 113]. A genome-wide association study also identified *TP63* (rs17505102) and *PTPRJ* (rs920590) as germline susceptibility loci in childhood t(12;21) ETV6-RUNX1 pre-B ALL [114]. Around 15% of t(12;21)+ patients harbour an activation mutation in *NSD2*, which could lead to an increase in H3K36 dimethylation [115]. Further studies also confirmed that t(12;21) ETV6-RUNX1 pre-B ALL is associated with hypermethylation compared to other subtypes such as t(1;19) E2A-PBX1, which has been associated with hypomethylation in the same study [116]. Interestingly, ETV6-RUNX1+ patients also show a preserved global H4 acetylation, which is a favourable prognostic factor [117]. A recent study described a group of patients with an *ETV6*-*RUNX1*-like gene expression signature, some of whom also harboured deletions in *ETV6* and *IKZF1* [118]. This paper additionally identified a new subset of paediatric pre-B ALL patients harbouring a *DUX4*-rearrangement. This leads to an overexpression of DUX4, a homeobox-containing protein, and a loss of function of the *ERG* transcription factor [119].

### ETV6 and RUNX1: transcription factors vital for embryonic and adult HSCs

In 1997, Wang et al. published the generation of an *ETV6* knock-out mouse line [120], with the complete knock-out resulting in embryonic lethality at E10.5 due to an absence of angiogenesis in the yolk sac and apoptosis in specific intra-embryonic cell types. In 2010, Ciau-Uitz et al. showed in Xenopus embryos that Etv6 is essential for the specification of HSCs and the arterial identity of the dorsal aorta through the regulation of Vegfa expression [121]. This study places ETV6 as an essential cell-extrinsic regulator of HSC emergence. In adult blood cells, Runx1 is expressed in transplantable HSCs, myeloid cells and in some lymphoid cells [122], but there is an absolute requirement for the presence of Runx1 only during the initial generation of HSCs during embryogenesis [37]. The lack of Runx1 does not affect yolk sac erythropoiesis, but it severely compromises definitive haematopoiesis [123].

In 1998, Wang et al. investigated the contribution of *Etv6* ^−/−^ embryonic stem cells in mouse chimeras [124]. They found that Etv6 is dispensable for foetal liver haematopoiesis, but crucially required for adult bone marrow haematopoiesis from the stage of initial bone marrow colonisation. Specifically, they found that *Etv6* ^−/−^ ES cells do not contribute to adult myelopoiesis or erythropoiesis, and the formation of B cells is also impaired in the absence of Etv6. In 2004, Hock et al. modulated the expression of Etv6 in different blood cell types by crossing mice harbouring a conditional (floxed) *Etv6* allele with mice expressing *CD19*-Cre (B cell), *Lck*-Cre (T-cell) and *Gata1*-Cre (erythrocytes and megakaryocytes) [125]. They did not observe changes in the proportions of B and T cells when *Etv6* was excised specifically in B or T cells, but found a significant reduction in the frequency of megakaryocytes. Using *Mx1*-Cre, however, led to a total disruption of adult HSC activity, suggesting that Etv6 is essential for HSC survival, while haematopoietic progenitors further downstream are largely Etv6-independent [125].

In 2015, Rasighaemi et al. inhibited the expression of Etv6 during zebrafish development using two distinct morpholinos [126]. The authors noted changes in primitive and definitive haematopoiesis during embryonic development, notably a decrease in erythrocytes and myeloid cells alongside an increase in lymphopoiesis. Together, those studies highlight the essential role for ETV6 in both the emergence of HSCs during embryonic development (cell-extrinsically) and their maintenance in adult tissues (cell-intrinsically). In addition, they show that Etv6, at least in zebrafish, can affect lymphopoiesis, which is interesting given that the complete inactivation of ETV6 is a frequent genetic event in t(12;21)+ pre-B ALL.

### The biology of t(12;21) ETV6-RUNX1 pre-B ALL

The ETV6-RUNX1 fusion gene possesses the PNT domain of ETV6, which is a transcriptional repressor of the Ets family. The PNT domain is the protein interaction domain and recruits proteins like NCOR, SIN3A and histone deacetylases to inhibit transcription [127]. The use of HDAC inhibitors confirmed that the recruitment of histone deacetylases by the PNT domain was part of the leukaemogenesis process, and this treatment led to an accumulation of cells in the G0/G1 phase [128]. Importantly, it affects RUNX1 target genes. For example, ETV6-RUNX1 can inhibit the transcriptional activation of *MCSFR*, normally promoted by RUNX1 or repressed by ETV6, and this inhibition requires the RUNX1 and C/EBPa consensus sequences [129]. ETV6-RUNX1 was also shown to have a dominant-negative effect on ETV6-mediated transcription, with increasing amounts of ETV6-RUNX1 inhibiting the ETV6-mediated transcriptional repression of stromelysin-1 [130]. Other transcriptional targets of ETV6 include *SPHK1*, *PTGER4* and *CLIC5*, which can promote survival and proliferation of leukaemia cell lines [131, 132]. Interestingly, ETV6-RUNX1 can also activate the expression of RAC1, which phosphorylates and activates STAT3, leading to a constitutive activation of C-MYC [133]. Accordingly, the inhibition of STAT3 activity through specific inhibitors induced cell death in the pre-leukaemia cell, and shRNA directed against Stat3 led to a longer latency in pre-B ALL development.

Similar to t(4;11) MLL-AF4, many mouse models have been developed to assess the role of ETV6-RUNX1 in disease development. In 2001, Andreasson et al. published a transgenic mouse model, in which the human ETV6-RUNX1 fusion gene was inserted into the locus of the immunoglobulin heavy chain enhancer/promoter E_µ_V_H_P [134]. This promoter is active in pre-B cells, so the expression of ETV6-RUNX1 was initiated in the population of leukaemia blast cells observed in patients. The mice did not display any signs of haematological malignancy even though the expression of ETV6-RUNX1 was detected in the bone marrow and spleen. This is the first study to suggest that ETV6-RUNX1 alone is not sufficient to drive the development of pre-B ALL.

Bernardin et al. used a transduction approach to study the role of ETV6-RUNX1 in wild-type and Cdkn2a^−/−^ bone marrow cells, followed by transplantation [135]. The alpha form of Cdkn2a (p16_INK4a_) binds to cyclin-dependent kinase Cdk4 and Cdk6 to inhibit their kinase activity [136]. This leads to a lack of phosphorylation of pRB and a G1 arrest. The beta form of Cdkn2a (p19_ARF_) activates the p53-MDM2 pathway leading to a G1 cell cycle arrest in both G1 and G2/M [137]. Bernardin et al. found that mice developed an acute leukaemia with a similar penetrance in transduced wild-type and Cdkn2a^−/−^ cells, but the latency was shorter when Cdkn2a was deleted. Li et al. crossed a *Tie2*-Cre with an inducible ETV6-RUNX1 transgenic mouse, thus targeting expression to endothelial and haematopoietic cells. These mice were then bred to a Cdkn2a-null mouse to assess the contribution of Cdkn2a to leukaemogenesis. Mice that expressed ETV6-RUNX1 and lacked Cdkn2a developed pre-B ALL with a high frequency, and the latency was further reduced with irradiation [138]. Considering that Cdkn2a expression is lost in about 25% of t(12;21)+ pre-B ALL patients, both studies suggest that the inactivation of Cdkn2a is an event that contributes to accelerate leukaemia development [139].

Tsuzuki et al. used a similar approach to Bernardin et al. and transplanted bone marrow cells transduced with a retrovirus expressing ETV6-RUNX1 to investigate the first stages of disease development [140]. They found that ETV6-RUNX1 increased ckit+ haematopoietic stem and progenitor cells, but also increased the proportion of pre-pro-B and pro-B cells in the bone marrow. This B cell differentiation arrest was not complete since there were still pre-B and mature B cells being formed from ETV6-RUNX1+ bone marrow cells. The myeloid and erythroid compartments were largely unaffected. This study suggests that ETV6-RUNX1 can contribute to a differentiation arrest during B lymphoid development, but its presence alone is not sufficient for a complete arrest.

Similarly, Fischer et al. transduced early haematopoietic progenitors with ETV6-RUNX1 and investigated the B cell differentiation arrest and the incidence of leukaemia development in their cohort [141]. Even though their mice did not get sick, the ETV6-RUNX1 transduced cells formed more pre-B cells. When ETV6-RUNX1 expression was higher, the transduced cells started to express ckit and the CD11b myeloid marker. It is plausible that the fine-tuning of ETV6-RUNX1 expression is crucial for restricting the differentiation program towards the B lineage fate. This phenotype has lately been attributed to the transactivation domain of ETV6-RUNX1, which is part of the RUNX1 part of the fusion gene [142].

Similar to t(4;11) MLL-AF4 pro-B ALL, xenotransplantation has been used to assess the role of ETV6-RUNX1 in a human cellular context [143]. The CD34^+^CD38^−^CD19^+^ fraction of t(12;21) ETV6-RUNX1+ patient pre-B ALL bone marrow was transplanted into severe combined immunodeficient mice (NOD/SCID). This population was able to engraft and form B cell colonies that could serially replate. They were also resistant to apoptotic stimuli induced by camptothecin, Fas-L and melphalan.

Schindler et al. used a different approach and inserted the human *RUNX1* gene into the mouse *Etv6* locus to generate a fusion gene that, like in patients, is under the control of endogenous *Etv6* gene regulatory elements [144]. The expression of the Etv6-RUNX1 gene was activated with *Gata1*-Cre to target early haematopoiesis in the embryo. About two-thirds of the embryos survived an early lethality and showed no obvious phenotype apart from a mild B cell expansion even though foetal liver cells failed to reconstitute the lymphoid compartment upon transplantation. A failure of Etv6-RUNX1-expressing HSCs to differentiate down the lymphoid lineage was further confirmed using *Mx1*-Cre and *Vav*-Cre induction.

Kantner et al. created a mouse model of ETV6-RUNX1 by knocking the human ETV6-RUNX1 into the CD19 locus [145]. This led to the expression of ETV6-RUNX1 under the control of the CD19 promoter, which will target committed B lymphoid cells exclusively. The expression level of ETV6-RUNX1 was similar to ETV6, suggesting that it recapitulates the expression level found in leukaemia blasts from patients. The transgenic mice showed similar proportions of the Hardy B cell fractions along the differentiation pathway in the bone marrow and a slight, but not significant, increase of immature B cells in the spleen. Even though the mice did not get sick, pro-B cells showed an elevated cellular level of reactive oxygen species, which translated into an accumulation of DNA damage. This study unravelled a new function of the ETV6-RUNX1 gene as a mediator of DNA damage, a step necessary for disease progression.

Another transduction approach targeted immature (Lineage− or ckit+) cells or pro-B cells from the foetal liver to study the early changes conferred by the ETV6-RUNX1 fusion gene in leukaemogenesis [146]. One retroviral vector conferred high expression of ETV6-RUNX1 while the other vector conferred lower expression, similar to what is observed in patients. The cells with the lower expression of ETV6-RUNX1 could form more and bigger B lymphoid colonies, whereas high expression of ETV6-RUNX1 had a negative impact on B cell development. The ETV6-RUNX1 low-expressing cells also showed an enhanced replating potential, demonstrating that low expression of ETV6-RUNX1 conferred self-renewal to pro-B cells. Interestingly, this phenotype was specific to foetal cells and was not recapitulated in adult cells. Therefore, similar to what is reported for MLL-AF4, it is crucial to maintain an expression level similar to what is observed in patients and to initiate the expression of the fusion gene at the right stage of development.

**Fig. 2:**
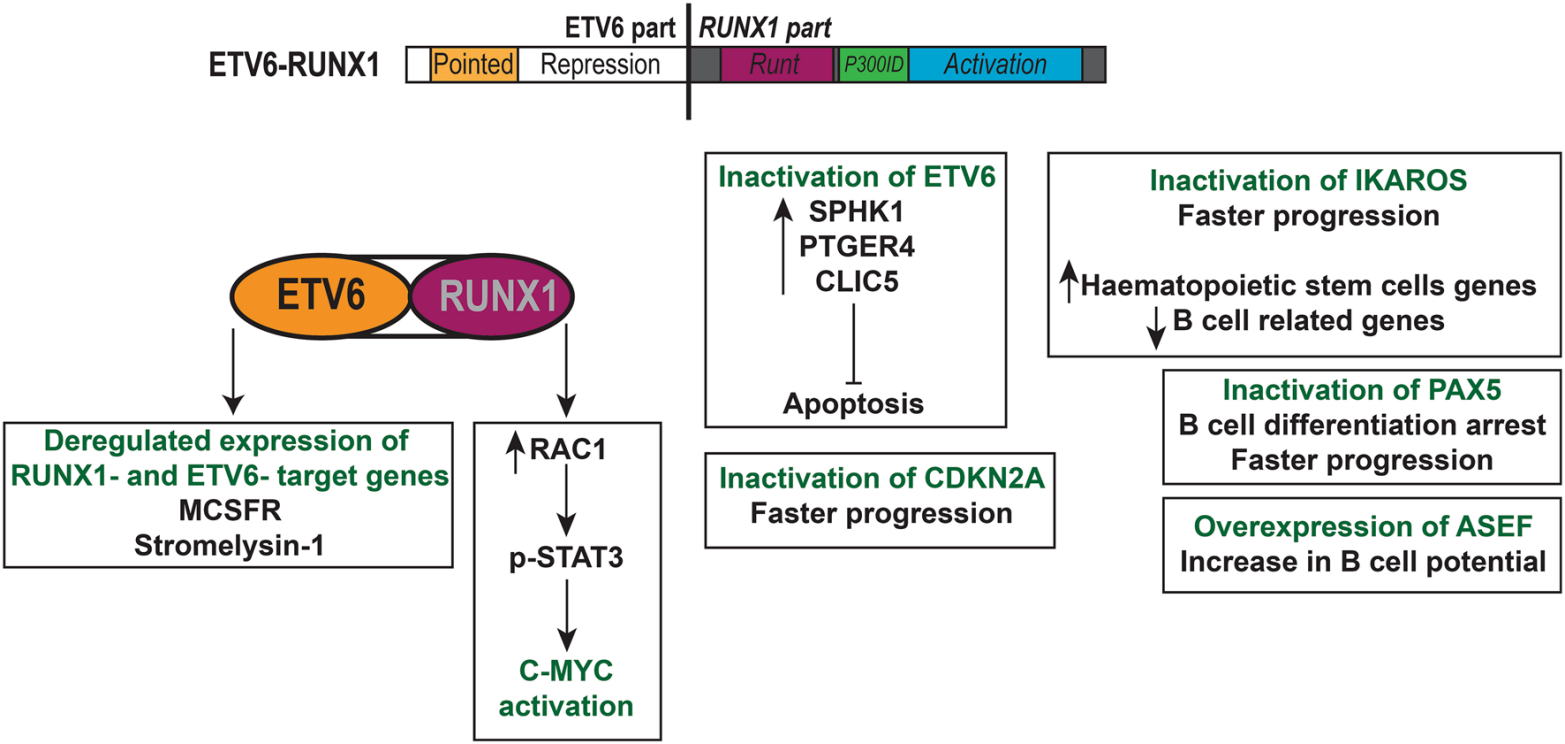
t(12;21) ETV6-RUNX1 leukaemia pre-B acute lymphoblastic leukaemia. The different parts of the *ETV6*-*RUNX1* fusion gene are depicted and include the repression domains of *ETV6* with almost the entire *RUNX1* gene. The *ETV6*-*RUNX1* fusion gene can alter gene expression by targeting RUNX1- and ETV6-target genes such as *MCSFR* and *stromelysin*-*1*. This leukaemia requires cooperating genetic mutations including the inactivation of *ETV6*, *IKAROS*, *PAX5*, *CDKN2A* and overexpression of *ASEF*. The prognosis is excellent and almost all patients are cured under the current therapy regimens

Several studies investigated the involvement of additional mutations in t(12;21) ETV6-RUNX1 leukaemogenesis. The *ASEF* gene, a RAC specific guanine nucleotide exchange factor, was reported to be overexpressed in t(12;21) ETV6-RUNX1 pre-B ALL, but not in other haematological malignancies [147]. Its overexpression led to increased B cell potential both in vitro and in vivo, but was not sufficient to induce pre-B ALL (not even in the presence of ETV6-RUNX1). To study t(12;21) in the context of accumulating mutations, van der Weyden et al. generated a mouse model that expressed the Etv6-RUNX1 fusion gene and the Sleeping Beauty transpose under the control of the *Etv6* promoter [148]. Around 20% of the mice developed pre-B ALL with a latency of approximately 1 year, which compared to the models mentioned previously and which can be attributed to the presence of additional mutations. By sequencing the genomic DNA from sick mice, they found mutations in genes that correlated with patient data, one of which was *Ikzf1* (Ikaros). The deletion of *Ikzf1* has been reported in pre-B ALL and leads to an increased expression of HSC genes and reduced expression of B cell genes [149]. It is essential for pre-B cell differentiation [150], so its inactivation would favour the differentiation arrest observed in pre-B ALL. The same group then replaced the Sleeping Beauty transposase component with a *Pax5* haploinsufficiency [151]. *PAX5* is often deleted or mutated in t(12;21) ETV6-RUNX1 pre-B ALL, with the mutations leading to decreased transcriptional activity [152]. Mice developed a transplantable B cell ALL that was similar to their previous model [148]. Both studies confirmed that t(12;21) ETV6-RUNX1 pre-B ALL requires additional mutations to progress to a full-blown leukaemia. Interestingly, Martìn-Lorenzo et al. also showed that the haploinsufficiency of *Pax5* alone can lead to the development of pre-B ALL when the immunological system of the mouse is challenged by an environment that contains pathogens [153]. Both the study by van der Weyden et al. and the one by Martìn-Lorenzo et al. suggested that somatic mutations in Jak3 could also facilitate leukaemia progression [151, 153]. One of the main oncogenic drivers in t(12;21) ETV6-RUNX1 pre-B ALL is RAG-mediated recombination [154]. In 2015, Swaminathan and Klemm et al. showed that genetic lesions in *ETV6*-*RUNX1*+ pre-B ALL are usually mediated by AID or RAG1 [155]. Their activity is also increased through chronic inflammation, highlighting the contribution of an overstimulation of the immune system to leukaemogenesis. Another study measured a ten-fold elevation of RAG1 in *ETV6*-*RUNX1*+ patients compared to other subtypes of pre-B ALL and found that AID was more expressed in patients lacking a cytogenetic change [156]. All these studies combined suggest that the expression of ETV6-RUNX1 leads to an arrest in the B cell differentiation, but the complete arrest and proliferation of leukaemia blasts requires additional genetic events such as the loss of Pax5 or Cdkn2a activity, which are potentially mediated by an elevated RAG-mediated recombination. The biological features of t(12;21) ETV6-RUNX1 pre-B ALL are summarised in Fig. 2.

## The other usual suspect: t(1;19)(q23;p13) E2A-PBX1

### Clinical features of E2A-PBX1+ pre-B ALL

The t(1;19) E2A-PBX1 translocation occurs in approximately 25% of paediatric pre-B ALL. It leads to the fusion of PBX1 exons containing the DNA-binding domain and the homeobox domain with the exons of E2A containing the transactivation domain [157]. Alternative splicing leads to the expression of five E2A-PBX1 proteins, where p85^E2A-PBX1^ and p77^E2A-PBX1^ are the dominant isoforms [158]. The transforming activity of both proteins is poor in vitro, but can lead to tumour formation in nude mice. Similar to other pre-B ALL, the leukaemia blast population in patients is CD10^+^CD19^+^CD34^+^ [94]. This subtype of leukaemia has been associated with a poor prognosis, but this is mostly for patients that have a balanced t(1;19) translocation [159].

### E2A is crucial for the maturation of B lymphoid cells

The *E2A* locus, also known as *TCF3*, expresses two proteins: E12 and E47. Once these helix-loop-helix proteins dimerise, they bind E-box sites in promoters and enhancers of genomic DNA to regulate gene expression [160]. E2A is an important regulator of B cell development. The knock-out of *E2a* in mice results in a high rate of post-natal death [161, 162]. The surviving embryos suffered from growth retardation and an absence of B lymphoid cells, due to reduced levels of Pax5 in the foetal liver. The remaining lineages (T cells, erythrocytes, granulocytes and macrophages) were unaffected by the knock-out of *E2a*. The expression of E2a peaks during the pro-B to pre-B cell transition once Scl is turned off, and is subsequently downregulated once IgM expression is turned on [163].

E2A activity is negatively regulated by serum growth factors and cell cycle entry, as well as by the ID proteins. The family of ID proteins has been implicated in cancer development and can inhibit the transcriptional activity of Ets transcription factors as well as members of the paired domain transcription factors such as Pax5 (as reviewed in Ref. [164]). The expression of ID2 and ID3 is significantly reduced in pre-B ALL compared to AML, suggesting that their presence is not favourable to pre-B leukaemia blasts [165]. Interestingly, the activity of E2a is also reduced in the bone marrow and spleen of aging mice [166], at a time when HSCs show a trend towards producing fewer lymphoid cells [167]. Overall, the modulation of E2A activity can lead to perturbation in B cell development, thus explaining its involvement in paediatric pre-B ALL.

### PBX1, a member of the Hox family of genes

PBX1 is a TALE-class homeodomain transcription factor that interacts with other members of the HOX family of genes to increase their DNA-binding specificities and affinities [168]. PBX1 is a DNA-binding partner of MEIS1 [169], which is also upregulated in t(4;11)+ MLL-AF4 pro-B ALL. The knock-out of *Pbx1* in mouse embryonic development leads to profound anaemia and death by E16 because of severe skeletal defects [170]. The absence of Pbx1 also results in abnormalities in the patterning of the great arteries and cardiac outflow tract during embryonic development [171].

In embryonic haematopoiesis, Pbx1 deficiency causes a decrease in myeloid colony-formation and a severe reduction in the engraftment of HSCs from the foetal liver [170]. Pbx1 is highly expressed on the ventral site of the E11.5 AGM, especially in the mesenchyme, but also in haematopoietic progenitors and individual endothelial cells. It continues to be expressed in foetal liver haematopoietic progenitors at later stages and is vital for maintaining definitive haematopoiesis. Pbx1 also plays an essential role in maintaining the quiescence of long-term adult HSCs and in lymphopoiesis [172, 173]. Therefore, the disruption of Pbx1 activity can compromise self-renewal and lymphoid potential of haematopoietic stem and progenitor cells.

### The biology of t(1;19) E2A-PBX1 pre-B ALL

The E2A-PBX1 fusion gene is a chimeric transcription factor that lost its ability to interact with MEIS1, but can still interact with HOX genes [174]. Whereas PBX1 transcriptional activity is normally low, E2A-PBX1 can constitutively activate the transcription of PBX1 targets [175]. The stability of the E2A-PBX1 fusion gene can be decreased by HDM2, which promotes ubiquitination and degradation, and increased through acetylation by GCN5 [176]. The interaction between E2A-PBX1 and GCN5 facilitates the expression of target genes such as WNT16 [177], a secreted signalling protein that can activate the expression of Notch ligands (DIc and DId) required for definitive haematopoiesis [178]. Similar to other B cell leukaemias, several mouse models have been developed to understand the role of E2A-PBX1 in leukaemogenesis.

In their 1993 manuscript, Kamps et al. transduced haematopoietic progenitors from the bone marrow with a retrovirus encoding p85^E2A-Pbx1^, one of the major isoforms, and transplanted these cells to assess leukaemia development [179]. The mice developed leukaemia 11–30 weeks post-transplantation and presented with splenomegaly and an infiltration of the liver and lungs with proliferating blasts. These blasts did not express markers of B or T-cells, but expressed myeloid markers, suggesting that the mice had developed AML, most of which could also grow as granulocytic sarcomas. Using a similar experimental scheme, Sykes et al. found that early pro-T cells immortalised by E2A-PBX1 can generate a myeloid leukaemia (AML) when injected into mice [180]. They also suggested a co-dependence between Scf and E2A-PBX1 for pro-T cell proliferation, which is similar to the co-dependence of SCF and MEIS1 in AML.

In 1993, Uckun et al. presented the results from xenotransplantations of human t(1;19) pre-B ALL leukaemia blasts into SCID mice [181]. The mice developed pre-B ALL within 7–12 weeks after transplantation, with a phenotype recapitulating the human disease. Interestingly, the engraftment was higher when the pre-B ALL blasts came from a patient that relapsed, whereas the pre-B ALL blasts from patients with no relapse did not engraft as well. This study highlights the relationship between the prognosis in patients and the engraftment ability of leukaemia blasts, suggesting the re-acquisition of stem-cell like features in poor prognosis pre-B ALL.

Dedera et al. generated a transgenic mouse model of t(1;19) E2A-PBX1 in which the expression of the fusion gene is under the control of the immunoglobulin heavy chain (IgH) variable region promoter fused to the IgH enhancer (Eµ) [182]. This targets the expression of E2A-PBX1 exclusively to lymphoid cells. Transgenic mice developed T cell leukaemia or lymphoma by 5 months of age. Before the onset of disease, a reduction in the number of thymocytes and bone marrow B lymphoid progenitors was observed. The remaining thymocytes were larger in size compared with cells from control animals, suggesting higher replication and proliferation, but they also exhibited a higher rate of cell death. This suggested that E2A-PBX1 can induce both proliferation and apoptosis simultaneously.

Through the inactivation of CD3 expression, Bijl et al. developed a mouse model which lacked immature and mature T cells [183]. E2A-PBX1/CD3ε^−/−^ mice developed a pre-B cell leukaemia with a latency of around 400 days that was accompanied by an activation of the Hox locus. This feature is also observed in t(4;11) MLL-AF4 pro-B ALL, and thus suggests that HOX activation is involved in another subtype of pre-B ALL.

Using the same mouse model, Hassawi et al. then investigated the role of Hoxa9 in t(1;19) E2A-PBX1 pre-B ALL [183, 184]. The overexpression of Hoxa9 promoted B cell proliferation and led to faster disease progression in E2A-PBX1/CD3ε^−/−^ mice. This was accompanied by reduced levels of Pax5 and Ebf1 gene expression, and an activation of Flt3. The same group also tested the role of HOXB4 in t(1;19) E2A-PBX1 pre-B ALL [185]. First, they performed retroviral transduction of B220^+^ cells derived from the bone marrow of E2A-PBX1/CD3ε^−/−^ mice and found that the expression of the *HOXB4* gene led to an increased production of pro-B cells in vitro, a phenotype linked to proliferation. These cells also exhibited a higher colony-forming potential compared with control cells. Second, they generated a HOXB4/E2A-PBX1/CD3ε^−/−^ mouse line in which HOXB4 and E2A-PBX1 were co-expressed in lymphoid cells. The latency of the disease was not significantly different between E2A-PBX1/CD3ε^−/−^ and HOXB4/E2A-PBX1/CD3ε^−/−^ mice, but the authors detected a higher number of leukaemia-initiating cells upon HOXB4 expression. These studies suggest that HOXA9 and HOXB4 can participate in B cell leukaemogenesis through different mechanisms.

In a recent xenotransplantation study with the 697 leukaemia cell line derived from a t(1;19)+ pre-B ALL patient [186], the aim was to investigate the role of autophagy in leukaemia development through rapamycin treatment which inhibits mTOR activity, since it is known that the activation of autophagy can downregulate E2A-PBX1 expression [187]. All mice developed pre-B ALL with splenomegaly and hepatomegaly, regardless of the rapamycin treatment. However, the mice that received a rapamycin inhibitor after transplantation (or received cells that had been treated with a rapamycin inhibitor before transplantation) had a longer survival period and showed decreased infiltration of leukaemia blasts in the liver. Therefore, the inhibition of the PI3K/AKT/mTOR pathway may assist in eradicating leukaemia blasts, and there is ongoing work aimed at developing an inhibitor that will be useful in the clinic (as reviewed in Ref. [188]).

In a more recent mouse model, Duque-Afonso et al. used a conditional *E2A*-*PBX1* transgenic mouse line in combination with *CD19* or *Mb1*-driven Cre recombinase to target B cells [189]. They also employed *Mx1*-Cre to induce the expression by polyI:C injection. All mice developed pre-B ALL characterised by a higher white blood cell count, reduced platelet and red blood cell counts in the peripheral blood and splenomegaly. B cell maturation was found to be blocked at the pro-B to early pre-B transition. One of the most recurrent secondary mutations in those mice affected the activation of the Jak/Stat pathway, which may represent another interesting therapeutic avenue. Similar to t(12;21) ETV6-RUNX1 pre-B ALL, *Pax5* haploinsufficiency also accelerated leukaemia onset.

Subsequently, the same group reported hyperphosphorylation of Plcγ2 in E2A-PBX1+ mouse leukaemia blasts from the same model [190]. Plcγ2 is a key enzyme in B cell receptor signalling [191]. This hyperphosphorylation was associated with an increase in proliferation and was mediated by Zap70 (zeta chain associated protein kinase), Lck (lymphocyte-specific tyrosine kinase from the SRC family) and Syk (spleen tyrosine kinase), which are downstream targets of the E2A-PBX1 fusion gene. SYK can also upregulate BCL6, a gene essential for the formation of polyclonal B cells and involved in pre-B cell self-renewal [192]. Leukaemia blasts (pre-BCR+ ALL) can thus hijack the pre-BCR signalling pathway to induce differentiation arrest and avoid clonal extinction, and reactivating this pathway may be an attractive therapeutic target (as reviewed in Ref. [193]). Therefore, they also assessed the clonogenic potential of pre-BCR+ and pre-BCR− mouse leukaemia cells in the presence of an LCK inhibitor (A-770041) or SYK inhibitor (P505-15). Encouragingly, both inhibitors led to a significant decrease in colony-forming activity in vitro and increased the survival in vivo, with pre-BCR+ leukaemia cells being more sensitive to A-770041 and P505-15 compared with pre-BCR− leukaemia cells. Pre-BCR+ leukaemia blasts are further characterised by a constitutive activation of the PI3K-AKT signalling pathway and SRC kinase, as well as a downregulation of STAT5 activity [194]. The activation of pre-BCR is more frequent in patients with PBX1 rearrangement and is rarely seen in patients with a MLL rearrangement, t(12;21) ETV6-RUNX1 or t(9;22) BCR-ABL1.

Very recently, Eldfors et al. reported promising results with idelalisib, a phosphatidylinositol 3-kinase delta (p110δ) inhibitor, in a drug-sensitivity assay with leukaemia blasts from a t(1;19)+ relapse patient [195]. The viability of the leukaemia cells was greatly reduced compared with cells from a healthy donor. It also seemed to be more specific to the E2A-PBX1 fusion gene since leukaemia cell lines harbouring other genetic rearrangements were not as sensitive to idelalisib. P110δ is important for maintaining PI3K activity in mature B cells [196], and since the expression of p110δ seems to be more specific to haematopoietic cells, idelalisib may have minimal off-target effects.

Aurora B kinases are overexpressed in many types of paediatric pre-B ALL, but their expression is even higher in t(1;19) E2A-PBX1+ patients [197]. Interestingly, leukaemia cell lines show a significant decrease in proliferation when the expression of Aurora B kinase is downregulated through shRNAs or LNA-oligos. In addition, primary leukaemia cells from patients are more sensitive to the inhibitor barasertib-HQPA when Aurora B kinases are highly expressed. This is due to an increase in histone 3 phosphorylation at Serine 10, which is heavily phosphorylated during metaphase (as reviewed in Ref. [198]), and results in an accumulation of cells in the G2/M phase of the cell cycle, unable to complete cell division.

**Fig. 3:**
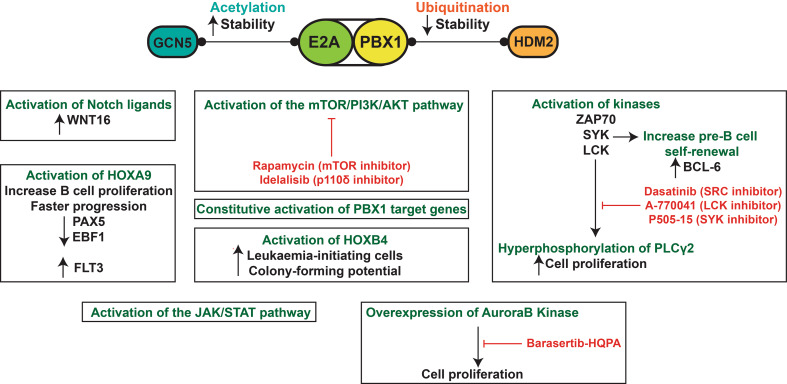
t(1;19) E2A-PBX1 pre-B acute lymphoblastic leukaemia. E2A-PBX1 can interact with GCN5 to increase its stability through acetylation or with HDM2 to initiate its degradation through ubiquitination. E2A-PBX1 can constitutively activate the expression of PBX1- target genes, which are expressed at low levels under physiological conditions. Many biological pathways contribute to t(1;19)+ pre-B ALL including the activation of Notch ligands, mTOR/PI3K/AKT, JAK/STAT, AuroraB kinase and the hyperphosphorylation of PLCγ2 through specific kinases. Potential therapeutic agents that target these pathways are shown in *red*

Rapamycin and its analogues are showing promising results in phase I and phase I/II clinical trials (NCT00874562 and NCT00081874), and a phase III clinical trial is completed for relapsed chronic lymphocytic leukaemia patients and shows that idelalisib improved their overall survival [199] (see also NCT01539512). This drug could also benefit B-ALL patients [195]. The LCK and SYK inhibitors are still in their preclinical phase. Phase I clinical trial for dasatinib (NCT00438854) and barasertib (NCT00926731, NCT01019161) are also completed. The biological features and therapeutic avenues of t(1;19) E2A-PBX1 pre-B ALL are highlighted in Fig. 3.

## The famous one: t(9;22) BCR-ABL1/Philadelphia chromosome

### Clinical features of t(9;22)+ leukaemia

The Philadelphia (Ph) chromosome is found in about 1–3% of paediatric pre-B ALL, and around 25% of adult patients [200]. BCR-ABL1 (or BCR-ABL) is also the main driver of chronic myelogenous leukaemia (CML) with a risk of blast-crisis [201, 202]. This chromosomal translocation leads to the fusion of the 5′ region of the BCR gene with the sequences upstream of the second exon of the ABL1 gene (almost the entire gene coding for this tyrosine kinase), giving rise to the p190 fusion protein found in approximately 85% of Ph+ paediatric ALL and 50–70% of Ph+ adult ALL [203, 204]. In adult CML, the fusion gene contains a larger portion of BCR, resulting in a 210 kDa fusion protein (p210). P210 can also be found in 15% of paediatric ALL and 30–50% of adult ALL [204]. Both p190 and p210 have constitutive kinase activity that can activate many pathways including RAS, RAC, PI3K/AKT/mTOR, NF-κB and JAK/STAT (as reviewed in Ref. [205]) [206]. P230 can be found in rare cases of CML and has a lower intrinsic kinase activity compared to p190 and p210 [204, 207]. Patients with CML are treated with the ABL1 kinase inhibitor imatinib mesylate, which ensures stable remission. Even though patients with pre-B ALL are less sensitive to tyrosine kinase inhibitors [208], the combination with standard chemotherapy has greatly increased the disease-free survival in both adults [209–211] and children [212, 213]. For example, the addition of imatinib increased the 4-year disease-free survival from 59% to 75% in paediatric ALL [214] (see also clinical trial NCT00025415). Recently, it was suggested that the ABL1 kinase inhibitor asciminib (ABL001), in combination with nilotinib, could eradicate the development of chronic myelogenous leukaemia in xenografts [215]. Both inhibitors can target the BCR-ABL1 mutant forms that are resistant to imatinib [216]. ABL001 is currently under clinical trial for t(9;22)+ ALL (NCT03106779). Similar to t(12;21) *ETV6*-*RUNX1* pre-B ALL, a genome-wide association identified a *GATA3* susceptibility locus (rs3824662) that increases the risk of ALL [217]. This locus is overrepresented in all patients, but even more in Ph+ ALL adolescents and young adults and relapsed patients [217, 218]. The deletion of *IKAROS* is another frequent genetic event in t(9;22) *BCR*-*ABL1* pre-B ALL [219]. A recent study by Witkowski et al. suggested that *Ikaros* has a tumour suppressor activity in Ph+ murine B-ALL and its inactivation is crucial for the maintenance of leukaemia [220].

### BCR: a breakpoint cluster region protein with kinase activity

BCR has an intrinsic serine/threonine kinase activity that is responsible for phosphorylating BCR serine and threonine residues [221, 222]. The BCR protein can also be phosphorylated by BCR-ABL1 in the part derived from the first exon [154]. The phosphorylation of Tyrosine 177 of BCR by FPS/FES protein tyrosine kinases leads to the binding of Grb2, an activator of Ras [223]. This interaction is important for p190 BCR-ABL1 leukaemogenesis through GAB2-SHP2 signalling [224]. The phosphorylation of Tyrosine 360 is important for the Bcr-mediated transphosphorylation of casein and histone H1, but also for inhibiting the serine/threonine kinase activity [225].

### ABL1: a non-receptor tyrosine kinase involved in B cell development

ABL1 is a member of the c-ABL kinase family and can be found in the cytoplasm as well as in the nucleus where it displays DNA-binding activity. The ablation of the C-terminal part of Abl in mice leads to a reduction in the B cell compartment, especially at the pro-B and pre-B stage [226]. The inhibition of c-ABL through a specific antisense oligonucleotide in CD34^+^ human bone marrow cells leads to an accumulation of cells in the G0/G1 phase of the cell cycle and a decrease in proliferation [227]. It also causes a significant loss in the formation of granulocyte-macrophage colonies. Abl was shown to activate JAK2 phosphorylation in haematopoietic cells [228], a pathway involved in many haematological malignancies. Similar to other genes involved in leukaemia, ABL1 is involved in haematopoiesis and B-lymphoid development.

### Biology of t(9;22) BCR-ABL1 pre-B ALL

In 1990, Heisterkamp et al. published a mouse model in which they used the metallothionein-1 promoter, which is active in most tissues, to express BCR-ABL1 [229, 230]. 60% of their transgenic mice developed an acute lymphoblastic leukaemia similar to paediatric pre-B ALL, while 20% developed a myeloid leukaemia which resembled the blast crisis phase of chronic myelogenous leukaemia. There was no thymus involvement for most animals, and the disease progressed rapidly within 10–58 days after birth. Interestingly, expression of BCR-ABL1 under the control of the BCR promoter leads to embryonic lethality [231].

The activation of the RAS pathway is a frequent feature of oncogenic transformation ([232], see also section on MLL-AF4). The human family of RAS comprises three members that are activated in many cancer types (NRAS, KRAS, HRAS) (reviewed in Ref. [233]). These small GTPases can bind and hydrolyse GTP, and their activation requires a series of post-transcriptional modifications to ensure localisation to the plasma membrane. The first of these is the addition of a farnesyl group to a conserved cysteine residue located in the CAAX motif of the C-terminal region. Without farnesylation, RAS remains in the cytoplasm and is inactive [234]. To determine if this was of therapeutic relevance, Reichert et al. investigated the effect of a farnesyl transferase inhibitor SCH66336, also known as lonafarnib, in the p190 BCR-ABL1 mouse model developed by Heisterkamp et al. [229, 235]. Mice that received SCH66336 had a significantly better survival (up to 250 days) than the vehicle control group or the no treatment group (~56 days). After termination of treatment, some mice developed leukaemia, suggesting that this drug cannot kill the leukaemia stem cells. The phase II clinical trial for lonafarnib is completed and could still benefit patients in combination with other drugs (NCT00034684).

The BCR-ABL1 fusion protein can interact with the p85α regulatory subunit of PI3K, an interaction vital for the proliferation of BCR-ABL1-dependent leukaemia cells [236]. The resultant activation of the PI3K/AKT pathway, which also requires the SH2 domain of BCR-ABL1, results in an upregulation of BCL-2 and C-MYC [237]. Accordingly, deletion of both Pi3k subunits (Pik3r1 and Pik3r2) impaired the transformation of murine bone marrow cells with p190 BCR-ABL1 and led to a loss of leukaemogenic potential [238]. An activation of the mTOR pathway independently of the PI3K/AKT pathway was also detected, suggesting that inhibiting both PI3K and mTOR (with the PI-103 inhibitor or similar), combined with the ABL inhibitor imatinib, could be a potential therapeutic avenue for Ph+ pre-B ALL. PI-103 never reached clinical trial because of its high in vivo metabolism [239], but alternative drugs are being tested in phase I clinical trials such as NVP-BEZ235 (NCT01756118).

In 2010, Henry et al. compared the effect of BCR-ABL1 on the B lymphoid potential of young and aged bone marrow cells [240]. Untransduced bone marrow cells showed a similar myeloid output, but the B lymphoid potential decreased with age. When transduced with p190 BCR-ABL1, cells of both ages displayed a higher engraftment and an expansion of early B cell progenitors, pro-B cells, immature and mature B cells when old bone marrow cells were used as competitor cells. Furthermore, p190 BCR-ABL1-expressing cells showed a higher level of phosphorylated Akt, Stat5 and Erk. The use of old competitor cells in their transplantation experiments, along with young or old bone marrow cells transduced with p190 BCR-ABL1, led to the development of leukaemia. Their study suggested that young competitor cells may not be ideal when modelling B cell leukaemia using transplantation.

In 2011, Notta et al. described a Ph+ ALL xenograft system that allowed the identification of multiple leukaemia-initiating clones in patients [241]. Recipients that were reconstituted with the predominant clone at diagnosis displayed a deletion of *CDKN2A/B* and fast disease. Interestingly, the xenograft clones constituted a population of leukaemia-initiating cells that did not always correspond to the sample at diagnosis. These results confirmed the heterogeneity of the leukaemia-initiating cell compartment and proposed that the engraftment is mostly due to more aggressive clones. This was also confirmed in another xenograft model by Ebinger et al. in 2016 [69].

Waldron et al. assessed the role of c-Myb and Bmi1 on the development of leukaemia in the p190 BCR-ABL1 mouse model [242]. They found that the loss of one c-Myb allele is sufficient to slow p190 BCR-ABL1 leukaemogenesis and that this is partly due to a decrease in Bmi1 expression. This study showed that activation of the c-Myb–Bmi1 axis is important for maintaining a high level of B-lymphoid clonogenic potential and ensuring disease progression of BCR-ABL1 pre-B ALL.

B cell ALL displays a differentiation arrest at the pro-B to pre-B stage and is associated with constitutive RAG activity, terminal deoxy-transferase expression and ongoing Ig heavy (IgH) chain rearrangement [243, 244]. In addition, the NF-κB transcription factor is constitutively active in ALL cells [245]. Ochodnicka-Mackovicova et al. investigated the relationship between the NF-κB and RAG pathways and noticed that the inhibition of NF-κB leads to an upregulation of RAG expression and increased RAG-dependent DNA damage in a transformed mouse pre-B cell model [246]. In patients, a low expression of RAG1, RAG2 and TDT correlates with higher expression of NF-κB. Since the inhibition of NF-κB can lead to increased RAG-dependent DNA damage in patient leukaemia cells, this therapeutic avenue should be considered with extreme care. Rag-mediated recombination can lead to the deletion of *Ikaros* in BCR-ABL1+ pre-B ALL, which facilitates leukaemogenesis [219].

**Fig. 4:**
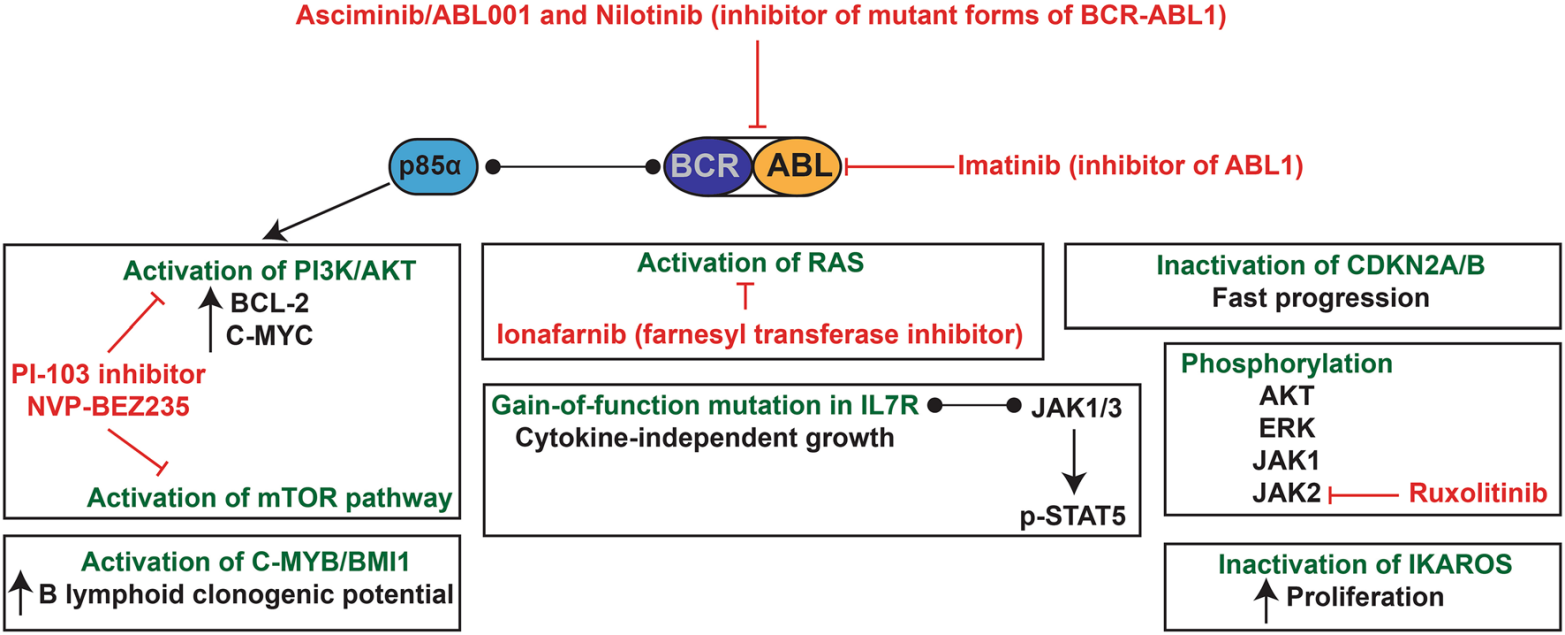
t(9;22) BCR-ABL1 pre-B acute lymphoblastic leukaemia. This leukaemia depends on the hijack of signalling pathways, including the constitutive activation of ABL kinase and the activation of PI3K/AKT/mTOR pathways. These can be targeted by inhibitors of ABL (imatinib), mutant forms of BCR-ABL1 (asciminib/ABL001 and nilotinib) and PI3K/mTOR (PI-103 and NVP-BEZ235). The activation of RAS, IL7R, C-MYB/BMI-1 and the phosphorylation of JAK2 can also contribute to disease. RAS can be inhibited by lonafarnib, a farnesyl transferase inhibitor, and ruxolitinib can inhibit JAK2 activity

The IL7 receptor (IL7R) is crucially involved in lymphoid development and its loss leads to reduced lymphoid cellularity in the peripheral blood [247]. Shochat et al. recently reported gain-of-function mutations in IL7R in paediatric B- and T-ALL through a serine-to-cysteine substitution (Ser185) in the extracellular region, or in-frame insertions and deletions in the transmembrane domain [248]. This resulted in the constitutive activation of the receptor and cytokine-independent growth of progenitor lymphoid cells. IL7R, once activated, can interact with JAK1 and JAK3 to phosphorylate and activate STAT5. The JAK/STAT pathway is often activated in leukaemia blasts of Ph+ pre-B ALL [249, 250]. P190 BCR-ABL1 can mediate the tyrosine phosphorylation of STAT members, and JAK1 and JAK2 have been found to be constitutively activated by mutations in the kinase and pseudokinase domains, which explains the sensitivity of Ph+ leukaemia cells to JAK2 inhibitors [251, 252]. The biological features and therapeutic avenues of t(9;22) BCR-ABL1 pre-B ALL are presented in Fig. 4.

### *BCR*-*ABL1*-like

In 2009, Den Boer et al. constructed a classifier based on gene expression to shed light on the 25% of pre-B ALL cases that are genetically unclassified [253]. This led to the identification of a *BCR*-*ABL1*-like phenotype in 15–50% of paediatric cases that harbour deletions in *IKZF1*, *PAX5*, *EBF1*, *TCF3* and *VPREB1*. The *BCR*-*ABL1*-like group was then confirmed in 30–40% of adult B-ALL cases [254]. In both age groups, this subtype is associated with a poor response to treatment through resistance to L-asparaginase and daunorubicin, and also a high risk of relapse [253]. In 2014, Roberts et al. published a genome profiling study aimed at identifying genetic alterations that could explain the poor prognosis [255]. They found a number of rearrangements of genes involved in tyrosine kinase signalling (*ABL1*, *ABL2*, *CRFL2*, *CSF1R*, *EPOR*, *JAK2*, *NTRK3*, *PDGFRB*, *PTK2B*, *TSLP* and *TYK2*) and mutations in *FLT3*, *IL7R* and *SH2B3*. Some of these rearrangements led to a constitutive activation of STAT5 and a sensitivity to dasatinib (*ABL1*, *ABL2*, *CSF1R* and *PDGFRB* fusion genes), ruxolitinib (*EPOR* and *JAK2* rearrangements) and crizotinib (*ETV6*-*NTRK3*) [255, 256]. Furthermore, the use of tyrosine kinase inhibitors in *BCR*-*ABL1*-like patients and refractory cases has shown promising therapeutic results [257–259]. The phase II clinical trial for dasatinib is completed (NCT00438854) while the recruitment has started for ruxolitinib (NCT02723994). *Ex vivo* analysis with primary patient cells are underway for crizotinib (NCT02551718).

### Hyperdiploidy

Around 20–30% of paediatric and around 3% of adult B cell leukaemia are associated with a gain in chromosome numbers. There is evidence for a prenatal origin in some patients, but the frequency is low compared to other subtypes such as t(4;11) MLL-AF4 or t(12;21) ETV6-RUNX1 [260]. Patients respond very well to therapeutic drugs or DNA topoisomerase inhibitors due to an overstimulation of the apoptotic response by hyperdiploid leukaemia cells [261, 262]. A significant proportion of patients harbours mutations in *FLT3* (~25%), *NRAS/KRAS* (~15%) or *PTNP11* genes (~10%), which are also involved in other paediatric pro- and pre-B ALL [263–265]. These mutations are mutually exclusive in most cases [266]. Other frequent abnormalities involve deletions in loci of *ETV6*, *CKDN2A*, *PAX5* and *PAN3 [* 267 *]*.

### Hypodiploidy

Hypodiploidy is a rare subset of B cell leukaemia in both children and adults (1–2%) and is associated with a very poor prognosis. The aetiology is not known, but the gene dosage or unmasking of recessive alleles could be part of the answer (as reviewed in Ref. [268]). Holmfeldt et al. recently described the genomic landscape of hypodiploid ALL using whole-genome and exome sequencing [269]. Around 71% of near-haploid patients (24–31 chromosomes) had mutations in genes leading to the activation of receptor tyrosine kinases and Ras signalling, and 13% presented deletions and one frameshift mutation in *IKZF3*, a lymphoid transcription factor of the IKAROS family. They also found mutations in *TP53* (~90%), *IKZF2* (~36%) and *RB1* (~41%) in low-hypodiploid patients (32–39 chromosomes). All patients showed activation of Ras- and PI3K-signalling and were sensitive to PI3K inhibitors, highlighting a potential therapeutic avenue. A phase I clinical trial is underway for the dual PI3K/mTOR inhibitor NVP-BEX235 (NCT01756118), and the phase I trial for the PI3K inhibitor GDC-0941 is complete (NCT00876122).

### Trisomy 4 and 10

Trisomy of chromosomes 4 and 10 accounts for 20–25% of paediatric pre-B ALL, but is very rare in adults (Table 1). It is associated with an extremely favourable 4-year event-free survival (~96%) compared to around 70% for patients with none or just one trisomy (~70%) [270].

### Intrachromosomal amplification of chromosome 21

The involvement of chromosome 21 in leukaemia is present in 2% of cases of paediatric and 11% of adult B-ALL cases (as reviewed in Ref. [271]). Furthermore, children with Down syndrome have a 10–12 fold higher chance of developing an acute leukaemia compared to children without Down syndrome (as reviewed in Ref. [272]). This can lead to an amplification of the Runx1 locus in a subset of patients [273]. It is associated with a poor outcome and requires more intensive chemotherapy (HR COG ALL protocols) [274].

## Conclusion

In this review, we aimed at providing an updated report of the field of B cell leukaemia with a particular emphasis on infant/paediatric ALL. In both paediatric and adult patients, the transformation process is usually initiated by a chromosomal translocation, creating a fusion gene with aberrant activity. Some fusion genes will interfere at the transcriptional level (MLL-AF4, ETV6-RUNX1, E2A-PBX1) whereas others will activate signalling pathways that promote oncogenesis (BCR-ABL1). The first stages of transformation will prime the cells to become pre-malignant (pre-leukaemia cells) by inducing a differentiation arrest during B cell development and the acquisition of stem cell features (e.g. upregulation of Hox genes). What we have learned from the numerous mouse models of pro/pre-B ALL is that additional events are necessary to boost the transformation process and bring those pre-leukaemia cells to induce a full-blown leukaemia. These events include genetic mutations that inactivate key players of B cell development (e.g. Pax5, Ikaros, Ebf1) or inhibitors of apoptosis (e.g. BCL-2 and MCL-1). There may also be an activation of genes involved in cell division and proliferation (e.g. CDK6, phosphorylation of PLCγ2). We are only starting to identify and understand the impact of co-drivers on pro/pre-B ALL, and these co-drivers can lead to the development of novel therapeutic avenues. For future studies, it will also be important to include external factors that can recapitulate the human environment. For example, an overstimulation of the immune response can lead to B cell leukaemia in Pax5 haploinsufficient mice, whereas a clean environment does not lead to disease. Similar findings could be true for other factors (e.g. pesticides). It is also essential to understand the role of these mechanisms specifically in leukaemia stem cells as relapse from these therapy-resistant cells is a major clinical problem.

## Abbreviations

AGM: Aorta-gonads-mesonephros
AML: Acute myeloid leukaemia
B-ALL: B cell acute lymphoblastic leukaemia
CLP: Common lymphoid progenitor
CML: Chronic myelogenous leukaemia
CMP: Common myeloid progenitor
ES: Embryonic stem
GMP: Granulocyte–macrophage progenitor
HSC: Haematopoietic stem cell
LMPP: Lymphoid-primed multipotent progenitor
MPP: Multipotent progenitor
polyI:C: Polyinosinic/polycytidylic acid
PreMegE: Premegakaryocytic/erythroid
VEC: VE-cadherin
